# Tumor loci and their interactions on mouse chromosome 19 that contribute to testicular germ cell tumors

**DOI:** 10.1186/1471-2156-15-65

**Published:** 2014-05-30

**Authors:** Rui Zhu, Angabin Matin

**Affiliations:** 1 Department of Systems Medicine and Bioengineering, Houston Methodist Research Institute, Houston, Texas 77030, USA; 2 Department of Genetics, The University of Texas M.D. Anderson Cancer Center, Houston, Texas 77030, USA

**Keywords:** Congenic strain, Chromosome substitution strain, M19, Testicular germ cell tumor, Modifiers, Epistasis

## Abstract

**Background:**

Complex genetic factors underlie testicular germ cell tumor (TGCT) development. One experimental approach to dissect the genetics of TGCT predisposition is to use chromosome substitution strains, such as the 129.MOLF-Chr 19 (M19). M19 carries chromosome (Chr) 19 from the MOLF whereas all other chromosomes are from the 129 strain. 71% of M19 males develop TGCTs in contrast to 5% in 129 strain. To identify and map tumor loci from M19 we generated congenic strains harboring MOLF chromosome 19 segments on 129 strain background and monitored their TGCT incidence.

**Results:**

We found 3 congenic strains that each harbored tumor promoting loci that had high (14%-32%) whereas 2 other congenics had low (4%) TGCT incidences. To determine how multiple loci influence TGCT development, we created double and triple congenic strains. We found additive interactions were predominant when 2 loci were combined in double congenic strains. Surprisingly, we found an example where 2 loci, both which do not contribute significantly to TGCT, when combined in a double congenic strain resulted in greater than expected TGCT incidence (positive interaction). In an opposite example, when 2 loci with high TGCT incidences were combined, males of the double congenic showed lower than expected TGCT incidence (negative interaction). For the triple congenic strain, depending on the analysis, the overall TGCT incidence could be additive or could also be due to a positive interaction of one region with others. Additionally, we identified loci that promote bilateral tumors or testicular abnormalities.

**Conclusions:**

The congenic strains each with their characteristic TGCT incidences, laterality of tumors and incidence of testicular abnormalities, are useful for identification of TGCT susceptibility modifier genes that map to Chr 19 and also for studies on the genetic and environmental causes of TGCT development. TGCTs are a consequence of aberrant germ cell and testis development. By defining predisposing loci and some of the locus interactions from M19, this study further advances our understanding of the complex genetics of TGCTs, which is the most common cancer in young human males.

## Background

Testicular germ cell tumors (TGCTs) are the most common cancers that afflict young men. Higher incidence of TGCTs in certain ethnic groups and in men with a family history of TGCTs [1-3] indicates a strong genetic predisposition to this cancer. Some of the genes, genetic loci and signaling pathways that contribute to TGCTs have been identified [4-9]. These data indicate that multiple genetic defects contribute to TGCT development and that individual genes contribute with relatively modest effects. Thus additional genetic defects that contribute to TGCT predisposition are yet to be identified.

Most TGCTs initiate in utero even though the disease becomes evident decades after birth. The majority of testicular tumors originate from germ cells [10,11]. In mice, TGCTs occur on the 129 strain background and resemble the prepubertal, pediatric TGCTs that occur in humans [12]. 5% of 129/Sv (129S1/SvImJ) males spontaneously develop TGCTs [13-16]. Defects in genes such as *DMRT1* or *KITLG* are associated with TGCTs in both humans and mice [9,17-20] indicating that some of the underlying genetic causes of TGCT development is shared among mice and humans.

The present work is further extension of earlier studies in which a genome-wide linkage scan analysis revealed that multiple loci from MOLF chromosome (Chr) 19 contributed to TGCT development [21,22]. This result was initially surprising because the MOLF strain is TGCT resistant and not known to harbor TGCT predisposing loci. However, to verify the linkage result, a consomic mouse strain was made, the 129. MOLF-Chr 19 (or M19) [21,22]. In M19, Chr 19 of 129 is replaced with that of MOLF whereas all its other chromosomes are from the 129 strain. It is known that males of the 129 mouse strain are inherently susceptible to develop TGCTs at a low frequency whereas the MOLF strain is TGCT resistant. However, 71% of M19 males developed TGCTs [16,22], which confirmed the linkage analysis results. The MOLF strain, and thus Chr 19 from MOLF, belongs to the subspecies *Mus musculus molossinus*, whereas 129 strain is *Mus musculus domesticus*[23]. Thus the M19 strain, derived from interspecific crosses, shows a more extreme, transgressive phenotype compared to its parental strains [24]. This indicates that genes from MOLF Chr 19, when placed on a 129 strain background, contribute to TGCT development. More specifically, polymorphisms between MOLF compared to 129 genes on Chr 19 likely aberrantly affect germ cell and testicular development resulting in high rate of TGCTs in M19. In support of the role of Chr 19 in TGCT development, 3 genes that map to Chr 19 have been shown to positively or negatively impact TGCT development. *Pten* deficiency increases TGCT incidence in mice [25]. *Dmrt1* deficiency increases TGCT incidence on the 129 strain background [19]. Thus, *Dmrt1* is a modifier, that is, it magnifies TGCT incidence of the 129 strain but does not cause TGCTs in all strain backgrounds. Decreased *Sf1* (Splicing factor 1) levels lowers TGCT incidence in mice [26]. Thus, the importance of mapping genetic loci as a first step towards the systematic identification of other genes on Chr 19 that contribute to TGCTs.

In an earlier study, we utilized a panel of congenic strains derived from the M19 strain to map TGCT loci on Chr 19 [16]. Most of the congenics carried large segments (7 congenics carried 12 to 34 cM segments and 3 congenics had less than 10 cM segments) of MOLF Chr 19 on the 129 background, and the MOLF segments in the different congenics overlapped to a great extent. Based on the tumor incidences of the congenics, we predicted that there are five MOLF-derived regions that harbor candidate TGCT modifier loci.

To further define the boundaries of TGCT modifier loci, in this study we generated congenic strains which carry smaller segments of MOLF Chr 19 and which lie within the predicted regions. Our results show that there are three TGCT susceptibility loci on Chr 19 that independently promote TGCT development. In addition, we find additive and epistatic interactions between loci that influence TGCT development in the M19. Interestingly, we found instances where 2 loci, both with either low or high TGCT incidences, when combined, contributed epistatically to higher or lower than expected TGCT incidences, respectively. Moreover, we found tumor loci that promote development of bilateral tumors (tumors that occur in both testes simultaneously) as opposed to loci that predispose tumor development in any one testis and loci that contribute to extremely high incidence of abnormal testes. Our results provide further novel insights into the complex genetics of TGCT development.

## Results

### Generation of congenic strains

**Figure 1:**
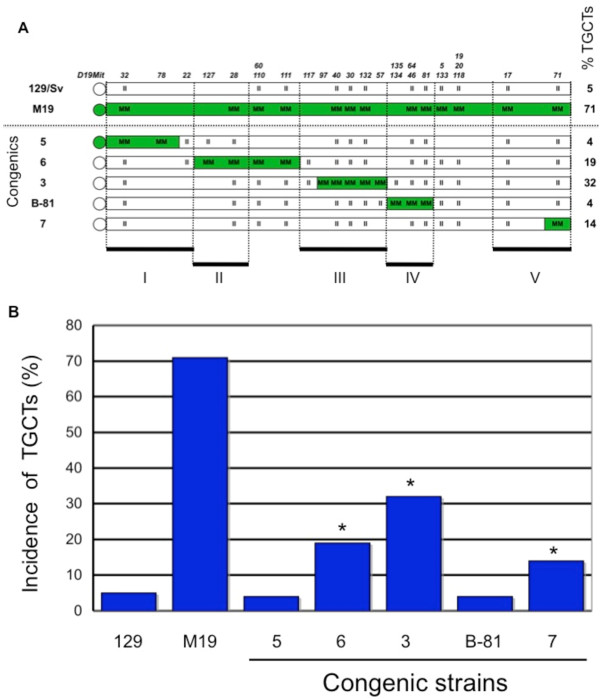
**Single congenics within regions I to V on mouse Chr 19. (A)** Representation of mouse chromosome 19 with centromere on left. Chr 19 of 129/Sv (represented in white), MOLF M19 (green) and MOLF segments (green) on 129/Sv background (white) in single congenic mouse strains. MOLF-derived segments are homozygous for MOLF alleles, represented by the MM genotype. 129-derived homozygous segments are represented by II genotype. Polymorphic SSLP markers between MOLF and 129 (starting at *D19Mit32* near the centromere) used for genotyping, are shown on the top. Names of each congenic strain are listed on the left. TGCT incidence of 129/Sv, M19 and congenics are on the right. The five predicted regions (I-V) are aligned with markers and shown at the bottom of the figure. **(B)** Bar graph represents TGCT incidences of 129, M19 and single congenic strains. *Indicates that TGCT incidence is significantly different from that of 129.

For an earlier study, we made congenic mouse strains that carried large segments, mostly 12–34 cM, of MOLF Chr 19 on a 129 background [16]. Based on the tumor incidences of these congenic strains, the data predicted the existence of 5 regions (regions I, II, III, IV and V) within Chr 19 that harbor TGCT susceptibility genes. The predicted regions I-V are indicated in Figure 1A. To test the predictions and to further map the extent of the TGCT susceptibility regions, we generated new congenic mouse strains that carry smaller (less than 10 Mb or 8 cM) MOLF Chr 19 segments on 129 background. The new congenic strains contain MOLF-derived segments that lie within Regions I, II and V and are named Congenic 5, 6 and 7. The MOLF segment in congenic 6 is slightly larger than predicted region II and in congenic 7 is smaller than predicted region V. Congenic 3 (also known as congenic-L1) which harbors 4.1 Mb (3.7 cM) region III [27] and B-81 with 1.4 Mb (3 cM) region IV [16] have been described previously and we include these strains in our analysis here.

The congenics 5, 6, 3, B-81 and 7 harbor consecutive regions of MOLF Chr 19 on the 129 strain background (Figure 1A). Thus, congenic 5 harbors a 4.3 Mb (2.3 cM) centromeric region of MOLF Chr 19 (from centromere to *D19Mit78)*, congenic 6 harbors a 9.4 Mb (7.1 cM) region of MOLF distal to that in congenic 5 (*D19Mit127* to *D19Mit111)*. Congenic 3 carries a 4.1 Mb (3.7 cM) region distal to congenic 6 (*D19Mit97* to *D19Mit57*) and congenic B-81 has 1.4 Mb (3 cM) MOLF region (*D19Mit135* to *D19Mit81)*. Congenic 7 contains a 1.7 Mb (0.6 cM) region of MOLF from *D19Mit71* to the telomere end of Chr 19. All congenics are homozygous for MOLF segments (represented as genotype MM) for the SSLP markers. Details regarding creation of congenics are described in the Methods Section. Because the previous study had indicated that TGCT susceptibility loci were not present between the SSLP markers *D19Mit5* and *D19Mit17* (10.2 Mb, 8.0 cM), this region is not represented in any new congenic strain [16].

### Three independent TGCT modifier loci are present on Chr 19

We determined the TGCT incidence of about 100 adult males from each congenic strain to evaluate the contribution of the MOLF-derived regions to tumor development. We noted the overall tumor incidence as well as the laterality of the tumors (unilateral tumor or bilateral testicular tumors) (Table 1). Our results showed that three of the five congenics had testicular tumor incidences higher than the expected background rate of tumor development found in the 129 strain (Figure 1B). The tumor incidences of the males of Congenic 6, 3, and 7 were 19% (*P* < 0.01), 32% (*P* < 0.0001) and 14% (*P* = 0.062), respectively. In contrast, the tumor incidences of the males of Congenic 5 and B-81 were both 4%. (TGCT incidences of congenic 3 and B-81 have been reported previously [16,27]). A TGCT incidence of 4% is similar to that of the 129/Sv strain (5%) and is considered a ‘background’ rate. The higher than background rate of TGCTs in congenics 6, 3 and 7 indicate that loci encompassing regions II, III and V harbor MOLF-derived TGCT predisposing genes which increase tumor incidence when present on the 129 strain background. Overall, these congenic strains clearly define three TGCT susceptibility, or modifier, loci that contribute to tumorigenesis.

**Table 1:** Incidence of testicular tumors and testicular abnormalities in the congenic strains

**Mouse strains**	**% TGCTs**	**No. with TGCT**	**% (no.) unilateral TGCTs**	**% (no.) bilateral TGCTs**	**% (no.) abnormal testes**	**No. of males examined**
*129	5%	4	5% (4)	0	15% (12)	83
*M19	71%	85	39% (46)	33% (39)	5% (6)	119
*Single congenics*						
5	4%	4	3% (3)	1% (1)	24% (23)	96
6	19%	25	16% (21)	3% (4)	26% (35)	134
*3	32%	26	21% (18)	10% (8)	9% (7)	82
*B-81	4%	5	4% (5)	0	10% (13)	129
7	14%	18	12% (16)	2% (2)	5% (7)	133
*Double congenics*						
5 × 3	34%	47	26% (35)	9% (12)	12% (17)	137
5 × B-81	11%	14	9% (12)	2% (2)	16% (21)	133
5 × 7	8%	11	8% (10)	1% (1)	14% (18)	130
6 × B-81	10%	9	10% (9)	0	15% (13)	88
1	22%	38	20% (35)	2% (3)	6% (10)	171
3 × 7	26%	32	18% (22)	8% (10)	11% (13)	122
*Triple congenic*						
5 × 3× 7	37%	50	30% (40)	7% (10)	8% (11)	136

*Previously described congenic strains [16,27].

### Negative and positive interactions between congenic regions

Next we examined whether the different regions of MOLF Chr 19 interact to additively increase TGCT incidence. The tumor incidences of congenic 6, 3 and 7 add up to 55% (see Methods for calculation), that is significantly less than the 71% TGCT incidence of M19 (*P* < 0.05). Thus, one possibility is that additional gene interactions likely account for the high TGCT incidence of M19. Alternately, although we followed the predictions of regions I-V to generate the new congenic strains, genes that map outside the MOLF segment boundaries and are not represented in the new congenics may affect TGCT susceptibility. In any case, to examine genetic interactions between the different regions, we combined the regions to create double and triple congenic regions and monitored TGCT incidences. First, we analyzed for interactions between two regions by calculating for expected additive effects and examining for any deviations from expected. A deviation from expected is considered an epistatic effect (interaction deviation) [28,29].

#### Primary interactions of region I (congenic 5)

**Figure 2:**
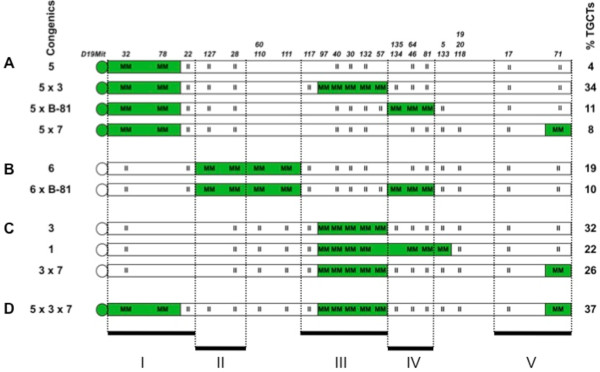
**Single and double congenic strains. (A)** Chr 19 of congenic 5 and derived double congenic strains 5x3, 5xB-81 and 5x7. MOLF or 129-derived segments are in green and white, respectively. Homozygous MOLF and 129 alleles are indicated as MM and II, respectively. TGCT incidences of the congenics are on the right. **(B)** Chr 19 of congenic 6 and derived double congenic strains. **(C)** Chr 19 of congenic 3 and derived double congenic strains. **(D)** Chr 19 of triple congenic strain 5 × 3 × 7.

**Figure 3:**
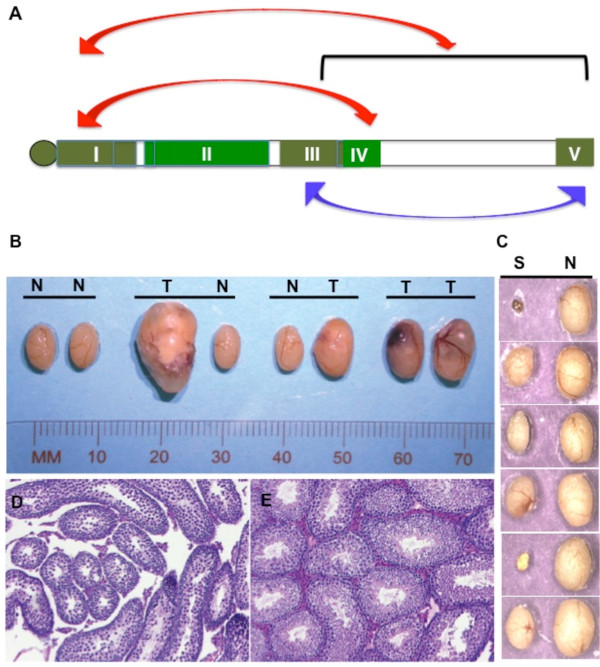
**Epistatic interactions and testes abnormalities. (A)** Epistatic interactions between regions. Bidirectional red arrows indicate positive epistatic interaction between regions I and IV and interaction of region I with (III.V). Bidirectional blue arrow indicates negative interaction between regions III and V. **(B)** TGCTs in congenic 3 mice. Specimens from left to right are: normal (N) pair of testes; tumor (T) in left testis and normal (N) right testis; normal left and tumor in right-testis; bilateral tumors in testes. **(C)** Abnormal testes from congenic 5 mice. Abnormal or small left testes (S) and normal (N) right testis. **(D)** Histological section of a small testis and **(E)** contralateral normal testis from congenic 5 mouse. In the small testes, spermatogenesis is arrested at specific stages. Few elongated spermatids are seen in the tubules.

To test the interaction of region I with other loci, we generated the double congenics, 5 × 3 (meaning that the strain carries both congenic 5 and congenic 3 segments, homozygous for MOLF, in regions I and III), 5 × B-81 and 5 × 7 (Figure 2A and Additional file 1: Table 2). We found that the TGCT incidence of congenic 5 × 3 (34%) was not significantly different from an expected additive effect of harboring regions I and III. The same was true for congenic 5 × 7 (Additional file 1: Table 2). Thus region I interacts additively with either regions III or V to increase TGCT incidences. In contrast, the double congenic strain 5 × B-81 had an elevated TGCT incidence (11%, *P* < 0.02) compared to the expected TGCT incidence (3%). This indicates a synergistic positive epistatic interaction between regions I and IV (Figure 3A). Curiously, individually both regions I and IV (represented in congenics 5 and B-81, respectively) have low TGCT incidences (4% each).

**Table 2:** Analysis for additive interactions between regions

**Region**	**Congenics**	**Observed incidence**	**Expected incidence**	**Test score (χ2,** ***P*-value)**
***Region I***				
I	5	0.04		
I.III	5 × 3	0.34	0.31	0.42, ns
I.IV	5 × B-81	0.11	0.03	5.96, *P* < 0.02
I.V	5 × 7	0.08	0.13	1.44, ns
***Region II***				
II	6	0.19		
II.IV	6 × B-81	0.10	0.18	2.28, ns
***Region III***				
III	3*	0.32		
III.I	5 × 3	0.34	0.31	0.42, ns
III.IV	1	0.22	0.31	3.37, ns
III.V	3 × 7	0.26	0.41	5.95, *P < 0.02*
***Region IV***				
IV	B-81*	0.04		
IV.I	5 × B-81	0.11	0.03	5.96, *P* < 0.02
IV.II	6 × B-81	0.10	0.18	2.28, ns
IV.III	1	0.22	0.31	3.37, ns
***Region V***				
V	7	0.14		
V.I	5 × 7	0.08	0.13	1.44, ns
V.III	3 × 7	0.26	0.41	5.95, *P < 0.02*

*Previously described congenic strains [16,27]; ns = no statistically significant difference between observed and expected values. Positive epistatic interactions are in green and negative epistatic interactions are in red.

#### Primary interactions of region II (congenic 6)

In the double congenic 6 × B-81 (combining regions II and IV), the 10% tumor incidence can be accounted due to additive interactions between regions II and IV (Figure 2B and Additional file 1: Table 2).

#### Primary interactions of region III (congenic 3)

Region III, in congenic 3, has the strongest independent effect on TGCT incidence compared to other regions (32% TGCT frequency). Region III interacts additively with either regions I or IV to increase TGCT incidences in congenic 5 × 3 and congenic 1, respectively (Figure 2A,C and Additional file 1: Table 2). However, there is negative epistatic interaction of region III with region V resulting in lower than expected TGCT incidences (26% observed as opposed to 41% TGCT expected due to additive interaction, *P* < 0.02) in the double 3 × 7 congenic strain (Figure 2C). Interestingly, individually both regions III and V (represented in congenics 3 and 7, respectively) have high TGCT incidences (32% and 14%, respectively).

#### Primary interactions of region IV (congenic B-81) and region V (congenic 7)

The double congenics 5 × B-81, 6 × B-81, 1, 5 × 7 and 3 × 7 that test interactions of region IV and V, have been discussed above (Figure 2 and Additional file 1: Table 2). The positive interaction of region IV with region I (in congenic 5 × B-81) and the negative interaction between regions V and III (congenic 3 × 7) have been described. Additive interactions account for increased TGCT incidences of the other double congenics.

#### Interactions between multiple loci

When multiple regions were present in a congenic strain as in 5 × 3 × 7 (which harbors regions I, III and V), the tumor incidence of 5 × 3 × 7 can be explained by an additive effect of the three regions (Figure 2D and Additional file 2: Table 3). On the other hand, we also asked, does region I interact with regions III and V in congenic 5 × 3 × 7? The observed tumor incidence of congenic 5 (harboring region I) is 4% and congenic 3 × 7 (harboring regions III and V) is 26%. Using this observation, we find that region I does indeed show positive epistatic interaction with regions III and V [written as I (III, V)] as the observed TGCT incidence (37%) is higher than expected (25%, *P* < 0.04) (Additional file 2: Table 3 and Figure 3A). However, similar analysis for interaction of regions III with I and V [III (I, V)] and V with I and III [V (I, III)] show no unexpected interactions. Thus, positive epistatic interactions, between multiple regions in congenic strains, could contribute to higher than expected TGCT incidence and could be one reason for overall higher than expected incidence of TGCTs in M19.

**Table 3:** Analysis for interactions between multiple regions

**Congenic strains**	**Regions**	**Observed incidence**	**Expected incidence**	**Test score (χ2,** ***P*** **value)**
5 × 3 × 7	I.III.V^¶^	0.37	0.40	0.25, ns
5 × 3 × 7	I (III.V)^§^	0.37	0.25	4.41, *P* < 0.04
5 × 3 × 7	III (I.V)	0.37	0.35	0.06, ns
5 × 3 × 7	V (I.III)	0.37	0.43	0.98, ns
3 × 7	III.V	0.26		
5 × 7	I.V	0.08		
5 × 3	I.III	0.34		

^¶^I.III.V indicates analysis of additive interaction between regions I and III and V.

^§^I (III.V) indicates analysis of interaction between region I and combined regions III and V.

ns = no statistically significant difference between observed and expected values.

Positive epistatic interactions are in green.

### Loci promoting bilateral tumors

We evaluated the laterality of TGCTs in the panel of single, double and triple congenics (Additional file 3: Table 4). We compared the observed and expected unilateral and bilateral tumor incidences in each congenic and applied χ^2^ goodness-of-fit tests [16]. Two of the strains, congenics 5 and 3, showed significantly increased incidence of bilateral TGCTs (*P* < 0.04 and *P* < 0.03, respectively). This suggests that susceptibility loci for bilateral TGCTs are located within regions I and III. For example, in congenic 3, one out of every three affected males developed bilateral TGCTs. The double congenics which harbor region III, 5 × 3 and 3 × 7, also showed higher number of bilateral TGCT cases than would be expected. Although congenic 5 has a low TGCT incidence (4%), it was significant that 1 out of 4 males had bilateral TGCTs. In contrast, no bilateral tumors were observed when a similar sized cohort of 129/Sv and B-81 (both strains have 4%-5% TGCT incidence) were examined [16].

**Table 4:** Laterality of TGCTs in the congenic strains

**Congenic strain**	**No. of males examined**		**No TGCT**	**Unilateral left tumor**	**Unilateral right tumor**	**Bilateral tumors**	**Test score**	**Left/Right**
5	96	Observed	92	2	1	1	8.76, *P* < 0.04	1.5
		Expected	91.3	2.8	1.9	0.1		
6	134	Observed	109	17	4	4	7.36, ns	2.7
		Expected	105.8	20.2	6.8	1.3		
3	82	Observed	56	10	8	8	8.97, *P* < 0.03	1.1
		Expected	51.2	14.4	12.8	3.6		
7	133	Observed	115	13	3	2	3.97, ns	2.8
		Expected	113.6	14	4.7	0.6		
5 × 3	137	Observed	90	20	15	12	8.37, *P* < 0.04	1.2
		Expected	84.4	25.2	21.1	6.3		
5 × B-81	133	Observed	119	8	4	2	5.73, ns	1.6
		Expected	116.2	10.1	6.1	0.5		
5 × 7	130	Observed	119	7	3	1	3.38, ns	2.0
		Expected	118.5	7.6	3.7	0.2		
6 × B-81	88	Observed	79	9	0	0	0, ns	All left
		Expected	79.2	8.8	0	0		
1	171	Observed	133	26	9	3	0.63, ns	2.4
		Expected	132	27	9.9	2		
3 × 7	122	Observed	90	12	10	10	16.98, *P* < 0.001	1.1
		Expected	84	18.4	16	3.5		
5 × 3 × 7	136	Observed	86	33	7	10	6.34, ns	2.5
		Expected	80.5	37.9	12	5.7		

ns = no statistically significant difference. Higher than Expected levels of Observed bilateral tumors are indicated in green.

### Incidence of abnormal testes in the congenic strains

While examining for testicular tumors, we observed that frequently males of some congenic strains had abnormal testes (Table 1). These testes were not tumorous but were grossly abnormal in shape or size. Often the abnormality affected one testis only. Histological examination of these abnormal testes frequently showed abnormal tubules and germ cells (Figure 3B-D). In congenics 5 and 6, close to a quarter (24% to 26%) of the males had abnormal testes. The abnormalities include cryptorchidism (undescended testes), atrophic calcified testes, dissociated dead testes, agonadism (missing testes), and hypoplastic testes with abnormal spermatogenesis (Figure 3D). These abnormalities resemble human testicular defects [30,31] and our observations indicate that genes causing testicular abnormalities are localized in regions I and II of Chr 19. In other congenic strains, the incidence of abnormal testes ranged from 5% to 16%.

## Discussion

Our previous study [16] had predicted the boundaries of multiple TGCT loci, additive effects and interactions between loci as well as a role for epigenetic modifications that result in high TGCT incidence in M19. However, the congenic mouse strains used in the previous study carried large MOLF segments making it difficult to ascertain the location and effects of TGCT-susceptibility loci with precision. In the present study, we generated and used congenic strains that carry smaller MOLF-derived segments so as to be able to test the predictions and define the effects and interactions of TGCT susceptibility loci with greater precision.

We first evaluated the regions I, II, III and V, which were previously predicted to harbor TGCT loci, by generating new congenic strains that harbor MOLF-derived segments encompassing these regions. Our results show that 3 regions, II, III, and V (in congenic strains 6, 3 and 7) contribute to higher than background frequencies of TGCTs. It is possible that genes located at the boundaries of regions I and V, which are not represented as MOLF segments in congenics 5 and 7, may harbor TGCT susceptibility genes. That could be one reason why congenic 5 (with region I) has low TGCT incidence of 4%. However, our analysis of double congenic strains show that although congenic 5 has low TGCT incidence, its MOLF segment interacts positively with other regions suggesting that this MOLF segment maps a ‘silent’ region that under some circumstances can contribute to TGCT development. Congenic 7 has a 14% TGCT incidence, which is similar to the 16%-17% TGCT incidence predicted for region V [16]. This suggests that we have mapped this TGCT susceptibility loci to a 1.7 Mb region between *D19Mit71* to the telomere, in congenic 7.

When we analyzed for interactions between any two regions, we found that in most cases, tumor incidences were due to additive effects of two regions. This was as predicted previously [16]. However, we also detected 2 unexpected epistatic effects. Regions I and IV interacted in a positive manner and the tumor incidence in double congenic strain 5 × B-81 was higher than expected. This was surprising, because individually regions I and IV (in congenic strains 5 and B-81) have low TGCT incidences. Thus we name regions I and IV ‘silent regions’ in terms of promoting TGCTs. These ‘silent regions’ can interact with other regions to increase TGCT incidence. Interestingly, the presence of the ‘silent region’ IV, but not I, was suggested in our previous study [16]. Overall, this indicates that TGCT mapping studies should be careful not to disregard loci with low tumor incidences because such regions could contribute with epistatic effects. Interestingly, males harboring the ‘silent’ region I (in congenic 5) have higher incidence of testicular abnormalities and bilateral tumors, although the overall incidence of TGCT in this strain is low (4%). In an opposite example, regions III and V in double congenic strain 3 × 7 showed lower than expected TGCT incidence. Again this was surprising because individually both regions III and V have high TGCT incidences. Although we could only test double interactions of a limited number of regions, our results offer tantalizing insights regarding how widely separated regions on Chr 19 can interact additively or epistatically to affect tumor incidences.

When multiple regions were present within a congenic strain as in the triple congenic 5 × 3 × 7, the overall tumor incidence appears to be due to an additive effect. However, further analysis revealed that the tumor incidence could also be considered to be due to a positive interaction of region I (with inherently low TGCT incidence) with regions III and V. Again in this example, even though region I does not contribute significantly to TGCTs, this ‘silent region’ appears to be important for epistatic interactions.

Thus, positive and negative interactions and the positive effect of ‘silent regions’ likely contribute to the overall high incidence of TGCT in M19. At the molecular level, there are a number of reasons that could explain the unexpected positive or negative interactions between regions. For example, genes that have wide ranging effects such as transcription factors, splicing factors, miRNAs, etc., may account for the larger than expected increases or decreases in TGCT incidences when regions are combined. For example we identified *Sf1*[26], an alternate splicing factor from region I, that likely affects a broad range of targets in cells of the testes to modulate TGCT incidence. Thus, locus interactions likely reflect the overall cumulative effects of gene interactions between suppressors (such as *Sf1*) and enhancers of TGCT development (such as *Dmrt1*) that reside within the five regions mapped to Chr 19. However, most of the TGCT susceptibility genes from M19 are yet to be identified.

Human genes involved in germ cell development, testicular dysgenesis, infertility and TGCT development likely reside in the orthologous regions mapped on mouse Chr 19. For example, region III is orthologous to human 9p24.1-9p24.3 (Table 5). Human disease loci which map to Chr 9p24 include *DMRT1.* Variants of *DMRT1* have been shown to be associated with susceptibility to pediatric germ cell tumors as well as familial testicular germ cell tumors [20,32,33]*. Dmrt1* is essential for testes differentiation in vertebrates [34,35]. Deficiency of *Dmrt1* in humans results in XY sex reversal and gonadal dysgenesis [36-39]. In mice, *Dmrt1* null mutants on a C57BL/6 background develop severe testicular dysgenesis but develop TGCTs on a 129 background [19]. Human genes related to gonadal dysfunction have so far not been reported to map to the other regions. In mice, a previous expression analysis study detected 3 transcripts, *Sf1, D19Bwg1357* and *Cox15,* that were differentially expressed in the gonads of M19 compared to 129 [27]. *Sf1* maps to region I (congenic 5) and lowered *Sf1* reduces TGCTs [26], which may be one reason why congenic 5 shows low TGCTs incidence. However, each locus may harbor more than one candidate TGCT gene. *D19Bwg1357* and *Cox15* map to region III (congenic 3) and proximal to region V, respectively. The genetic effects of *D19Bwg1357* or *Cox15* on TGCT development are unknown.

**Table 5:** Orthologous regions in the human genome corresponding to the five regions in mouse Chr 19

**Regions in mouse Chr 19**	**Mouse SSLP markers**	**Orthologous regions in humans**
		
I	*D19Mit32-D19Mit22*	11q12.2-11q13.2
II	*D19Mit22-D19Mit117*	9q21.11-9q21.31
III	*D19Mit117-D19Mit135*	9p24.1-9p24.3
IV	*D19Mit57-D19Mit5*	10q23.1-10q23.32
V	*D19Mit17-telomere*	10q24.33-10q26.11

*Source:*http://www.ensembl.org.

In our earlier mapping study, loci encoding bilateral tumors were not detected [16]. We think that because the congenic strains harbored larger MOLF-derived segments harboring multiple regions, interactions among these regions likely masked the presence of loci encoding bilateral TGCTs. We found that loci for bilateral TGCT development are located in regions I and III. Our results indicate that unilateral and bilateral tumors are not caused by the same genetic factors. One possible scenario is that genes that cause development of unilateral or bilateral tumors affect primordial germ cells at different stages during germ cell migration. Germ cells are specified as a group of cells in the early embryo, which then proliferate and migrate through the embryo before splitting into two populations that each migrate towards the left or right genital ridges. Each genital ridge later develops into a testis. Genes causing bilateral tumors may be expressed and functional during early germ cell development and thus defects or polymorphisms in these genes would affect the entire germ cell population before they split to populate the left or right genital ridges. Thus, defects or disease-causing nucleotide polymorphisms in such genes is manifested as an excess of bilateral tumors. However, genes causing unilateral tumors may be expressed and function later during germ cell development, such as once germ cells have entered either the left or right genital ridges. Thus defects or polymorphisms in the late expressed genes likely manifests as unilateral tumors.

We observed that the TGCT frequency of the left testis is about 2-fold higher than that on the right (Additional file 3: Table 4). Most of the abnormal testes were also found on the left side, whereas the right-side was usually normal (Figure 3D). The reason why left testes are more susceptible to abnormalities or TGCT development in mice is unclear.

Our previous study [16] also detected a correlation between the length of MOLF-derived congenic segment in a strain and TGCT frequency, and that was suggested to be due to epigenetic modifications imposed by the 129 background on the MOLF-derived chromosome. However, when we examined whether MOLF-length is a significant predictor of TGCT frequency in our present study, we found only a moderate positive correlation (R = 0.42) between MOLF-length and TGCT frequency. The linear regression model of our current data suggests that MOLF-length is not a significant predictor of TGCT frequency (R^2^ = 0.18; *P* = 0.17) (Additional file 4: Figure S1). One reason for the discrepancy between the two studies could be that the previous study used a total of 13 strains in which MOLF segments spanned 3 cM to 52.6 cM [16] and thus a correlation between length of MOLF segment and TGCT frequency was readily evident. In contrast, for the purpose of the present study, we utilized 12 strains (single, double and triple congenics) which harbored smaller MOLF regions (ranging from 0.6 cM to 10.1 cM). We think that because this study only examines a smaller, limited size range of MOLF-segments, the correlation between MOLF-length and TGCT frequency is not apparent.

The congenics reported here carry short segments of MOLF Chr 19 (10 cM or less) and each has defined, quantitative incidences for TGCTs, bilateral tumors and testicular defects. Thus, these strains will be useful to study how exogenous agents can modulate TGCT incidences. As an example, congenic 3 has been used to study the role of radiation, endocrine disruptors and chemotherapeutic agents on TGCT incidences [40,41]. Moreover, detailed gene expression array analysis has identified a limited number of candidate TGCT genes that map to the different loci (data not shown) and future work will evaluate the contribution of these candidate genes to TGCT development and other testicular defects.

## Conclusions

In summary, we have mapped three susceptibility loci on mouse Chr 19 that independently increases TGCT incidence. We found 2 regions on MOLF Chr 19 that contribute to bilateral TGCTs and 2 proximal regions that contribute to a very high incidence of testicular defects. Our results show that multiple loci on mouse Chr 19 together with their additive, negative and positive interactions affect germ cell and testes development to contribute to TGCTs. This study validates the use of chromosome substitution strain M19 to experimentally dissect the complex genetics of TGCT predisposition and further advances our understanding of the complex genetics of TGCTs, the most common cancer in young males.

## Methods

### Mouse strains

The 129.MOLF-Chr 19 (M19) [22] and 129 (129S1/SvImJ; JR002448, Jackson Laboratory, Bar Harbor, ME, USA) strains have been described. Mice were maintained on a 12/12 hour light/dark cycle, and fed NIH-31 diet ad libitum.

### Creation of single, double and triple congenic strains

The single congenic strains were created as described [16,27]. Briefly the M19 strain was crossed to 129. Progeny were backcrossed to 129. Backcross progeny were genotyped for selected polymorphic SSLP markers that distinguish 129 and MOLF alleles, for example, marker *D19Mit127* and *D19Mit111* for generating congenic 6 (Figure 1A). Mice whose genotypes were contiguously heterozygous for MOLF and 129 within the selected region and homozygous for 129 outside the region, were selected for further breeding. These selected mice were intercrossed to generate mice homozygous for MOLF containing segments. To verify the boundaries of the congenic regions, progeny were genotyped with additional SSLP markers spanning mouse Chr 19. SSLP markers used for genotyping are shown in Figure 1A. In Figure 1A, the homozygous MOLF regions on Chr 19 in each congenic strain are shown in green. For example, in congenic 6, markers *D19Mit127* and *D19Mit111* (and other SSLP markers in between) are homozygous for MOLF, indicated by genotype MM. Markers *D19Mit22*, *D19Mit117* and others on Chr 19 are homozygous for 129 (indicated by genotype II) and are shown in white.Double congenic and triple congenic strains harboring regions homozygous for MOLF (MM) alleles (Figure 2) were made by intercrossing appropriate single congenic strains followed by selection for homozygous pairs of F2 mice for further breeding and expansion of colony. For example, to create 5 × 7 strain, we first crossed congenic 5 female (homozygous for MOLF region 5) with congenic 7 male (homozygous for MOLF region 7). A pair of F1 male and female progeny (now heterozygous for MOLF-derived alleles in congenic region 5 and 7) were intercrossed. The F2 pups were genotyped and selected to identify pairs of males and females that were homozygous for both congenic 5 and congenic 7 MOLF-derived regions. Once selected, these homozygous 5 × 7 congenic male and female pairs were allowed to breed to produce homozygous 5 × 7 progeny and TGCT incidence in male progeny was assessed.

The experimental protocol was approved and conducted in compliance with Institutional Animal Care and Use Committee (IACUC) standards at MD Anderson Cancer Center.

### Tumor characterization

4–8 weeks old adult males were sacrificed and their testes examined for tumors. Tumors are usually detected visually at that age. In cases where it was unclear, testes were preserved in 10% phosphate-buffered formalin, sectioned and stained prior to microscopic examination for small tumors.

### Statistical testing for interacting loci on TGCT susceptibility

χ^2^ goodness-of-fit tests were used to evaluate whether observed frequency of TGCT is statistically different from expected additive values. The expected additive TGCT frequencies were calculated based on the previously described equations [16]. A background, baseline TGCT incidence of the 129 strain of 5% was used. Briefly, the tumor incidence of a single congenic strain is the sum of the baseline effect and the effect of the MOLF congenic segment (QTL effect). Thus, a TGCT incidence of a single congenic of 10% = 5% (baseline) + 5% (QTL/due to MOLF congenic segment). The expected tumor incidence for double and triple congenic strains is the sum of the QTLs (due to congenic segments) plus the baseline effect. Thus the expected tumor incidence of combining 3 congenic regions (with QTL1, QTL2 and QTL3 of 10%, 15% and 20% respectively) in one mouse strain will be 35% (=5% QTL1 + 10% QTL2 + 15% QTL3 + 5% baseline). Thus expected additive incidence in mice with 2 QTLs (2 congenic regions) is calculated as: (observed QTL1-baseline) + (observed QTL2-baseline) + baseline. Similarly, the expected additive incidence of 3 QTLs (3 congenic regions) = (observed QTL1-baseline) + (observed QTL2-baseline) + (observed QTL3-baseline) + baseline. As one example, to estimate additive effects of region III with V in congenic 3 × 7, the TGCT incidences of congenic 3 (32%), congenic 7 (14%) and 129 background TGCT incidence of 5% was used. Therefore, the expected additive incidence is [(32%-5%) + (14%-5%) + 5%] = 41%. Because the observed TGCT incidence of congenic 3 × 7 (26%) is significantly lower than 41% (*P* < 0.02), this indicates that region III interacts epistatically with region V (Additional file 1: Table 2).

Interactions between multiple regions (Additional file 2: Table 3) were calculated in a similar manner but regarding multiple regions as one. For example, to test possible interaction of region I with regions III and V in congenic 5 × 3 × 7, the TGCT incidences of congenic 5 (4%) and congenic 3 × 7 (26%) were used. Thus, the expected additive incidence of TGCTs by combining region I with III and V in [I(III.V)] is 25% = [(4%-5%) + (26%-5%) + 5%]. Because the observed TGCT incidence of congenic 5 × 3 × 7 (37%) differs significantly from 25% (*P* < 0.04), this indicates that region I interacts epistatically with regions III and V (Additional file 2: Table 3).

### Statistical testing for effects on tumor laterality

The expected frequencies of unaffected, unilateral or bilateral TGCT cases in the congenic strains were calculated, as described in [16]. χ^2^ goodness-of-fit tests were performed to determine if bilateral TGCTs were occurring randomly or caused by distinct factors on Chr 19.

## Abbreviations

TGCTs: Testicular germ cell tumors; Chr: Chromosome; M19: 129.MOLF-Chr 19; SSLP: Simple sequence length polymorphism; cM: Centimorgan; Mb: Megabase.

## Competing interests

The authors declare they have no competing interests.

## Authors’ contributions

RZ designed and performed the experiments, analyzed the data and wrote the manuscript. AM conceived the idea, designed the experiments, analyzed the data and wrote the manuscript. All authors read and approved the final manuscript.

## Supplementary Material

Additional file 1: Table S1 Analysis for additive interactions between regions. Click here for file

Additional file 2: Table S2 Analysis for interactions between multiple regions. Click here for file

Additional file 3: Table S3 Laterality of TGCTs in the congenic strains. Click here for file

Additional file 4: Figure S1 Scatter plot of the length of MOLF-congenic segments (Mb) versus TGCT frequencies. The linear regression line: y = 1.485x + 8.704 is shown. (Residual standard error = 11.15 on 10 degrees of freedom; multiple R-squared = 0.178; adjusted R-squared = 0.09575; *P*-value = 0.172.). Click here for file
