# Supplementary material for: Tumor loci and their interactions on mouse chromosome 19 that contribute to testicular germ cell tumors
Source: BMC Genet. 2014 May 30;15:65. doi: 10.1186/1471-2156-15-65 (PMC4053281; doi:10.1186/1471-2156-15-65)
Supplement: Additional file 1: Table S1 — Analysis for additive interactions between regions. [file 1471-2156-15-65-S1.doc]

**Additional file 1: Table S1** Analysis for additive interactions between regions

| Region | Congenics | Observed  incidence | Expected  incidence | Test score  (χ2, P - value) |
| --- | --- | --- | --- | --- |
| Region I |  |  |  |  |
| I | 5 | 0.04 |  |  |
| I.III | 5x3 | 0.34 | 0.31 | 0.42, ns |
| I.IV | 5xB-81 | 0.11 | 0.03 | 5.96, P < 0.02 |
| I.V | 5x7 | 0.08 | 0.13 | 1.44, ns |
| Region II |  |  |  |  |
| II | 6 | 0.19 |  |  |
| II.IV | 6xB-81 | 0.10 | 0.18 | 2.28, ns |
| Region III |  |  |  |  |
| III | 3* | 0.32 |  |  |
| III.I | 5x3 | 0.34 | 0.31 | 0.42, ns |
| III.IV | 1 | 0.22 | 0.31 | 3.37, ns |
| III.V | 3x7 | 0.26 | 0.41 | 5.95, P < 0.02 |
| Region IV |  |  |  |  |
| IV | B-81 * | 0.04 |  |  |
| IV.I | 5xB-81 | 0.11 | 0.03 | 5.96, P < 0.02 |
| IV.II | 6xB-81 | 0.10 | 0.18 | 2.28, ns |
| IV.III | 1 | 0.22 | 0.31 | 3.37, ns |
| Region V |  |  |  |  |
| V | 7 | 0.14 |  |  |
| V.I | 5x7 | 0.08 | 0.13 | 1.44, ns |
| V.III | 3x7 | 0.26 | 0.41 | 5.95, P < 0.02 |

*previously described congenic strains [16, 27] ; ns = no statistically significant difference between observed and expected values. Positive epistatic interactions are in green and negative epistatic interactions are in red.
