# Supplementary material for: Tumor loci and their interactions on mouse chromosome 19 that contribute to testicular germ cell tumors
Source: BMC Genet. 2014 May 30;15:65. doi: 10.1186/1471-2156-15-65 (PMC4053281; doi:10.1186/1471-2156-15-65)
Supplement: Additional file 2: Table S2 — Analysis for interactions between multiple regions. [file 1471-2156-15-65-S2.doc]

**Additional file 2: Table S2** Analysis for interactions between multiple regions

| Congenic strains | Regions | Observed incidence | Expected incidence | Test score  (χ2, P value) |
| --- | --- | --- | --- | --- |
| 5x3x7 | I.III.V ¶ | 0.37 | 0.40 | 0.25, ns |
| 5x3x7 | I (III.V) § | 0.37 | 0.25 | 4.41, P < 0.04 |
| 5x3x7 | III (I.V) | 0.37 | 0.35 | 0.06, ns |
| 5x3x7 | V (I.III) | 0.37 | 0.43 | 0.98, ns |
| 3x7 | III.V | 0.26 |  |  |
| 5x7 | I.V | 0.08 |  |  |
| 5x3 | I.III | 0.34 |  |  |

¶ I.III.V indicates analysis of additive interaction between regions I and III and V.

§ I (III.V) indicates analysis of interaction between region I and combined regions III and V.

ns = no statistically significant difference between observed and expected values.

Positive epistatic interactions are in green.
