# Supplementary material for: Tumor loci and their interactions on mouse chromosome 19 that contribute to testicular germ cell tumors
Source: BMC Genet. 2014 May 30;15:65. doi: 10.1186/1471-2156-15-65 (PMC4053281; doi:10.1186/1471-2156-15-65)
Supplement: Additional file 3: Table S3 — Laterality of TGCTs in the congenic strains. [file 1471-2156-15-65-S3.doc]

**Additional file 3: Table S3** Laterality of TGCTs in the congenic strains

| Congenic  strain | No. of  males examined |  | No  TGCT | Unilateral  left tumor | Unilateral  right tumor | Bilateral tumors | Test Score | Left /Right |
| --- | --- | --- | --- | --- | --- | --- | --- | --- |
| 5 | 96 | observed | 92 | 2 | 1 | 1 | 8.76, P < 0.04 | 1.5 |
|  |  | expected | 91.3 | 2.8 | 1.9 | 0.1 |  |  |
| 6 | 134 | observed | 109 | 17 | 4 | 4 | 7.36, ns | 2.7 |
|  |  | expected | 105.8 | 20.2 | 6.8 | 1.3 |  |  |
| 3 | 82 | observed | 56 | 10 | 8 | 8 | 8.97, P < 0.03 | 1.1 |
|  |  | expected | 51.2 | 14.4 | 12.8 | 3.6 |  |  |
| 7 | 133 | observed | 115 | 13 | 3 | 2 | 3.97, ns | 2.8 |
|  |  | expected | 113.6 | 14 | 4.7 | 0.6 |  |  |
| 5x3 | 137 | observed | 90 | 20 | 15 | 12 | 8.37, P < 0.04 | 1.2 |
|  |  | expected | 84.4 | 25.2 | 21.1 | 6.3 |  |  |
| 5xB-81 | 133 | observed | 119 | 8 | 4 | 2 | 5.73, ns | 1.6 |
|  |  | expected | 116.2 | 10.1 | 6.1 | 0.5 |  |  |
| 5x7 | 130 | observed | 119 | 7 | 3 | 1 | 3.38, ns | 2.0 |
|  |  | expected | 118.5 | 7.6 | 3.7 | 0.2 |  |  |
| 6xB-81 | 88 | observed | 79 | 9 | 0 | 0 | 0, ns | All left |
|  |  | expected | 79.2 | 8.8 | 0 | 0 |  |  |
| 1 | 171 | observed | 133 | 26 | 9 | 3 | 0.63, ns | 2.4 |
|  |  | expected | 132 | 27 | 9.9 | 2 |  |  |
| 3x7 | 122 | observed | 90 | 12 | 10 | 10 | 16.98, P < 0.001 | 1.1 |
|  |  | expected | 84 | 18.4 | 16 | 3.5 |  |  |
| 5x3x7 | 136 | observed | 86 | 33 | 7 | 10 | 6.34, ns | 2.5 |
|  |  | expected | 80.5 | 37.9 | 12 | 5.7 |  |  |

ns = no statistically significant difference. Higher than expected levels of observed bilateral tumors are indicated in green.
