# Supplementary figures and images for: Tumor loci and their interactions on mouse chromosome 19 that contribute to testicular germ cell tumors
Source: BMC Genet. 2014 May 30;15:65. doi: 10.1186/1471-2156-15-65 (PMC4053281; doi:10.1186/1471-2156-15-65)

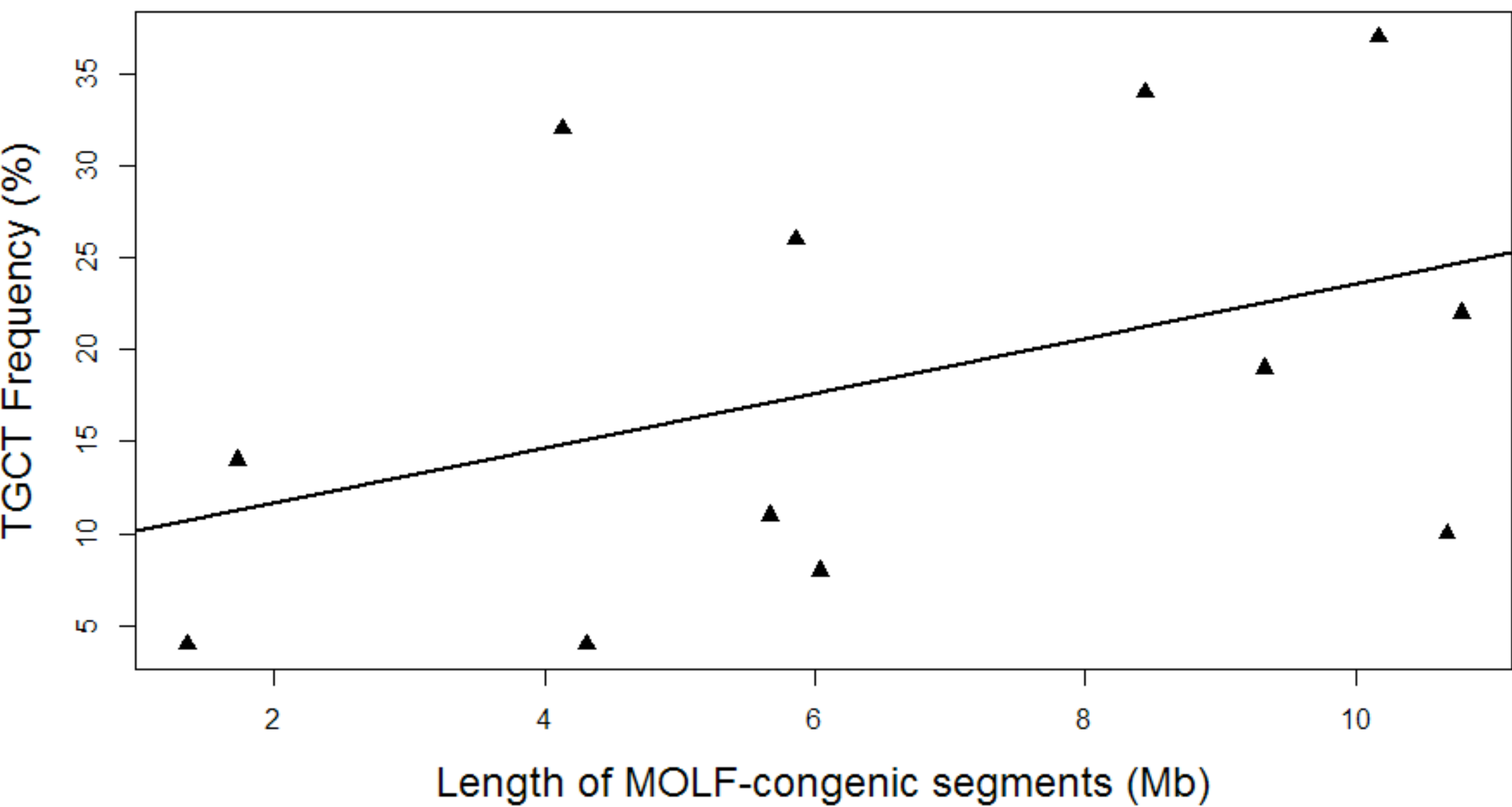

Supplement: Additional file 4: Figure S1 — Scatter plot of the length of MOLF-congenic segments (Mb) versus TGCT frequencies. The linear regression line: y = 1.485x + 8.704 is shown. (Residual standard error = 11.15 on 10 degrees of freedom; multiple R-squared = 0.178; adjusted R-squared = 0.09575; P-value = 0.172.). [file 1471-2156-15-65-S4.pdf]
